# Tumor Biomarker In-Solution Quantification, Standard Production, and Multiplex Detection

**DOI:** 10.1155/2021/9942605

**Published:** 2021-09-01

**Authors:** Nicole C. Japp, Joshua J. Souchek, Aaron R. Sasson, Michael A. Hollingsworth, Surinder K. Batra, Wade M. Junker

**Affiliations:** ^1^Sanguine Diagnostics and Therapeutics, Inc., Omaha, Nebraska, USA; ^2^Department of Surgery, Renaissance School of Medicine, Stony Brook University, Stony Brook, New York, USA; ^3^Eppley Institute for Research in Cancer & Allied Diseases, University of Nebraska Medical Center, Omaha, Nebraska, USA; ^4^Department of Biochemistry and Molecular Biology, University of Nebraska Medical Center, Omaha, Nebraska, USA

## Abstract

The diagnosis and monitoring of cancer have been facilitated by discovering tumor “biomarkers” and methods to detect their presence. Yet, for certain cancers, we still lack sensitive and specific biomarkers or the means to quantify subtle concentration changes successfully. The identification of new biomarkers of disease and improving the sensitivity of detection will remain key to changing clinical outcomes. Patient liquid biopsies (serum and plasma) are the most easily obtained sources for noninvasive analysis of proteins that tumor cells release directly and via extracellular microvesicles and tumor shedding. Therefore, an emphasis on creating reliable assays using serum/plasma and “*direct, in-solution*” ELISA approaches has built an industry centered on patient protein biomarker analysis. A need for improved dynamic range and automation has resulted in the application of ELISA principles to paramagnetic beads with chemiluminescent or fluorescent detection. In the clinical testing lab, chemiluminescent paramagnetic assays are run on automated machines that test a single analyte, minimize technical variation, and are not limited by serum sample volumes. This differs slightly from the R&D setting, where serum samples are often limiting; therefore, multiplexing antibodies to test multiple biomarkers in low serum volumes may be preferred. This review summarizes the development of historical biomarker “standards”, paramagnetic particle assay principles, chemiluminescent or fluorescent biomarker detection advancements, and multiplexing for sensitive detection of novel serum biomarkers.

## 1. Introduction

The demand for new serum biomarkers is driven by a need for improved detection and monitoring of cancer patient treatment/relapse. A genetic study of pancreatic tumors and paired metastatic tumors estimates that it may take between 10 and 16 years for a tumor-initiating cell to develop into metastatic subclones [1, 2]. Evidence suggests that most types of cancer, other than skin cancer, grow undetected for more than a decade and are discovered when a tumor starts causing symptoms or becomes visible on an X-ray or MRI [3]. Therefore, early detection may be improved if robust methods and sensitive biomarkers can be identified to detect the disease during its progression. The tumor biomarker clinical diagnostic industry uses bioinformatics tools, mathematical models, and enzymatic assays to identify and detect biomarkers and model a dynamic tumor microenvironment. The goal is then to identify biomarkers that can be reliably detected in serum at low numbers of tumor cells (<10^7^ cells) to improve clinical outcomes and patient care [4]. Clinicians often use biomarker levels to monitor response to treatments tailored to patients for personalized cancer therapy [5]. Clinical diagnostics are therefore heavily pursued and desired yet require complete biomarker characterization and complex assay validation. This is a large undertaking that requires many patient samples for validation and a comprehensive understanding of many disciplines. The principles Beckman Coulter and Luminex have developed exemplify the novel approaches for biomarker detection that have been applied in clinical diagnostics and the research and development industry.

## 2. Biomarker Detection

**Figure 1:**
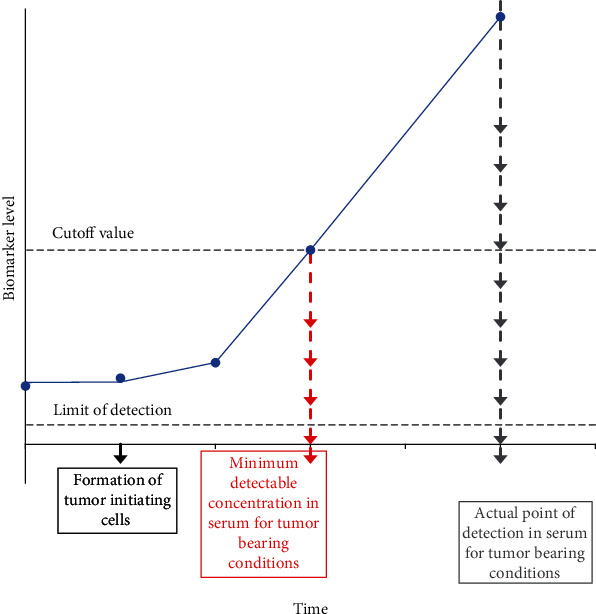
Minimum detectable concentration versus actual point of detection. The minimum detectable concentration (MDC) is the earliest detection point for a biomarker that exceeds its normal level of expression. As biomarkers are secreted by both healthy and tumor cells, the average biomarker level can be above the assay's detection limit before tumor initiation. Therefore, a cutoff value is required to differentiate between tumor-bearing and unaffected or benign conditions in serum assays. As tumor size increases, biomarker expression increases (blue circles). Although a biomarker is detected at or below the cutoff value, samples are not considered diseased until they exceed this cutoff value (the actual point of detection).

Serum-based assays are considered noninvasive tests as they monitor biomarker expression in the blood. Tumors and healthy cells both secrete proteins into circulation, but the serum of unaffected or benign conditions may express a lower level of protein as compared to that of patients with tumors [3, 6]. Thus, the expression of a critical biomarker is expected to increase as tumor growth occurs, and biomarker level cutoff points are required to identify tumor-bearing from unaffected individuals (Figure 1) [3]. However, most diseases do not express a single specific and sensitive biomarker and thus will require identification of a multiparameter panel to improve detection. These biomarker panels often contain proteins with varying levels of expression, which necessitates an assay with a wide dynamic range. Historically, the radioimmunoassay (RIA), enzymatic immunoassay (EIA), and enzyme-linked immunosorbent assay (ELISA) have provided a quantitative analysis of antigen in an unknown sample. RIAs have been replaced by EIA and ELISA due to the risks associated with hazardous radioactive elements. ELISAs have long been considered a “gold standard” in biomarker detection. Its principles are used in the recent advancement the diagnostic industry has made to improve upon its dynamic range.

Biomarkers are expressed in benign and malignant conditions; therefore, careful considerations must be taken when determining cutoff values to minimize the risk of false-positive results. Determining cutoff values is a tedious task, requiring biomarker-oriented or outcome-oriented models. A biomarker-oriented model separates a continuous marker by a mean biomarker value or percentile and implements the Gaussian distribution to produce a bimodal-shaped distribution to determine the optimal cutoff value [7]. An outcome-oriented model requires a correlation between binary variables to incorporate biomarker significance with the outcome (disease or no disease) and survival [8]. A training set of known clinical outcome is used to construct a receiver operating characteristic (ROC) curve and demonstrate the efficacy of a diagnostic biomarker using the finalized analytical assay. The optimal cutoff value is then determined by minimizing the Euclidean and Manhattan distance between points [8]. The area under the curve (AUC) depicts a biomarker's ability to separate cases from the control group(s). Multiple biomarkers may increase the combined power of detection as compared to a single biomarker and involve ROC curve analysis to assess the sensitivity (SN) and specificity (SP) of detection at different cutoff values [9]. As the developed analytical assay (method) directly influences biomarker detection and the realized SN/SP, the accuracy of biomarker detection and quantification may improve over time by applying advancements or new technology through the development of new biological standards reducing sources of error [10].

## 3. Historical Review of Biological Standards/Calibrator Production

The development of a biological standard requires careful considerations to accurately quantify biomarker levels in patient samples. Ideally, the standard's composition (matrix) is similar to that of human serum to account for the “matrix effect”, which may interfere with the assay's ability to detect an analyte and can result in inaccurate quantification of the biomarker. However, avoiding this phenomenon is often not fully considered by the vast majority of research use only (RUO) kit manufacturers, which may introduce error/bias during the quantification of a biomarker. Validation of a standard or reference material requires considerable work and is often just as important as the biomarker assay itself. Standards can be produced from a variety of starting materials (i.e., tumor cells, cell culture supernatant, cell culture total protein lysate, recombinant proteins, or synthetic peptides) and are purified using a wide range of techniques (i.e., gel filtration, affinity chromatography, or size exclusion chromatography). Other considerations that should be addressed during standard development include stability, storage temperature, and the preservative system. Beckman Coulter, Fujirebio Diagnostics, and Roche Diagnostics are established in the biomarker industry and produce FDA-approved standards (that are classified as antibody-defined tumor markers) for CA19-9, CEA, and CA125 (MUC16) clinical assays [11].

A thorough review of the literature was conducted to determine methods that were used in the production of CEA, CA19-9, and CA125 antibody-defined standards. CEA was isolated from an autopsy liver metastasis from primary carcinoma of the sigmoid colon. Perchloric acid precipitation and Sepharose 4B gel filtration were used to produce the “First British Standard for CEA 73/601” in 1975 [12]. CA19-9 was isolated from SW1116 cell culture supernatant. Reduction and alkylation (6 M guanidine hydrochloride) was followed by two rounds of affinity chromatography and produced a single (210 kDa) band in the presence of detergents, which were reassociated to form aggregates (600-2000 kDa) [13]. CA125 (MUC16) was identified in various ovarian cell line supernatants and was isolated from condensed OVCA 433 (serous cystadenocarcinoma cell line) supernatant. Perchloric acid precipitation, neutralization, dialysis to concentrate, molecular size exclusion on Sepharose CL-4B, and size exclusion on Sepharose CL-6B proceeded immunoaffinity purification with an OC125-Protein A-Sepharose CL-4B column. These approaches utilize antibody-specific reactivity for the basis of quantification and determination of units.

For antibody-defined tumor markers, the same starting material can be used for primary and secondary standards/calibrators [14]. An assay calibrator could in fact be the same material as the “secondary standard” but often is one step past and is defined by comparison back to a secondary standard that is carefully guarded as an internal manufacturing control at the “company”. It follows that the signal and quantification in an ELISA kit are dependent not only on (1) the reactivity of the antibody pair included in the kit but also upon (2) the preparation of the “calibrant” used to generate the standard curve. Thus, the apparent lack of industry standardization between different manufacturer's RUO kit offerings is due to the fact that there are rarely true reference assays or reference preparations to standardize against. Therefore, it is entirely possible that two different suppliers may produce kits (with different antibodies) for the same antigen that would result in different quantifications if used against the same standard curve (calibrant) material. Conversely, if two kits provide the same antibody-defined assay (same antibodies and methods), different results are possible if the standards/calibrants provided by each do not perform equally. A third complication is that even using the same calibrator and assay can produce different results with time. This is due to the instability of the calibrators that are in native forms. Variability in the quality of the calibrator preparation and changes with time and storage condition (degradation) can all affect performance (US20070141710A1-stable calibrators for immunoassays) [15].

The World Health Organization (WHO), companies, and academic researchers have worked together to produce “standards” of defined properties that are quantified with a reference method. These “reference preparations” or “standards” can be requested to standardize a “secondary standard” that can serve as an internal manufacturing control (“standard”) for the production of a kit calibrator or an automated immunoassay calibrator containing the same antigen. The “calibrator” value is usually therefore based on a “secondary standard”, whose value was first derived from the “reference preparation” or an accepted “standard”. There are limited offerings of reference standards. Complete lists can be obtained at WHO and NIBSC (National Institute for Biological Standards and Controls) repositories [16, 17].

### 3.1. Preparation of a MUC4/MUC5AC Mucin Protein Standard/Calibrator

Currently, there are no deposited reference standard preparations available for the mucin proteins MUC4 or MUC5AC, which are important biomarkers for pancreatic cancer, or for the heavily utilized CA19-9 biomarker that occurs on mucin proteins and is approved for monitoring of gastrointestinal (GI) malignancies. Suppliers of CA19-9 clinical assays denote in their 510(k) that their calibrators are matched to their “internal reference standard”. Therefore, to provide antibody-defined units for MUC4 and MUC5AC and promote biomarker research for pancreatic cancer, we have produced a new standard/calibrator material that will be used to provide “standardized” MUC5AC and MUC4 units in relation to the established CA19-9 units, for which secondary standards/calibrators can be obtained (i.e., from Beckman Coulter, Fujirebio Diagnostics, or Roche Diagnostics). Based on our historical review of tumor antigen/standard production, we reasoned size exclusion chromatography would be the simplest method for copurification of large glycoproteins. A cell line with high expression of MUC4 and moderate levels of MUC5AC and CA19-9 was used as the basis for our mucin standard. All preparatory steps for the growth/isolation/production of our standard have been determined and provide a copurified source of MUC4, MUC5AC, CA19-9, or “mucin-rich fraction” (MRF) that elutes from size exclusion chromatography. This serves as the “primary reference standard”, from which future calibrant lots can be calibrated to. The next step in finalizing our “primary” and “secondary” standards, as the first-ever MUC4/MUC5AC reference material, is to set “antibody-defined” units of the mucin core proteins MUC4 and MUC5AC. MUC4 and MUC5AC units will be set using the approach described for DU-PAN-2 [18]. This method includes setting the value of each serially diluted standard based on antibody reactivity with purified antigen.

## 4. Colorimetric-Based ELISA Approach

**Figure 2:**
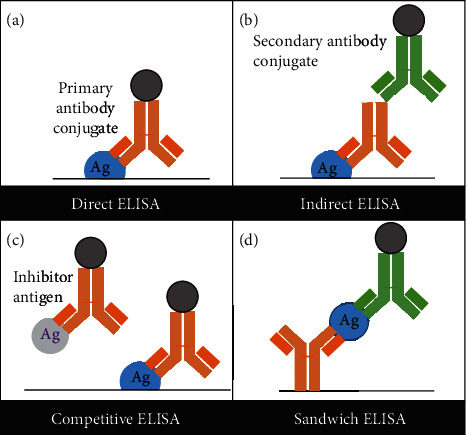
Types of ELISA. (a) Direct: an antigen is immobilized on the surface of a multiwell plate. A labeled primary antibody binds to the target antigen and is detected using an enzymatic substrate. (b) Indirect: an indirect ELISA consists of an unconjugated antibody binding to the target antigen, followed by a conjugated antibody. (c) Competitive: this assay is also known as an inhibition assay. The target antigen is precoated on a multiwell plate. An enzyme-labeled antibody is preincubated with the sample and may form antibody-antigen complexes with the inhibitor antigen before being added to the multiwell plate. The free antibody binds to the target antigen immobilized on the surface of a multiwell plate. A lower signal corresponds to a higher amount of antigen. (d) Sandwich: the sandwich “capture” assay is the most complex but provides sensitive and highly specific detection using two antibodies that preferably bind to two different epitopes. The antigen binds to the capture antibody and is detected using a second “detection” antibody. A labeled secondary antibody is then used to produce a measurable signal.

There are four main types of ELISA: direct, indirect, competitive, and sandwich (Figure 2). Biological molecules, such as proteins, viruses, hormones, and peptides, are detectable in small quantities using ELISA (high sensitivity over 2-3 orders of magnitude). *in direct* ELISA, an antigen is immobilized on the surface of a multiwell plate, which can hamper reaction kinetics, impose mass transport limitations, and impart steric hindrance, and yet is a starting point for labeled, single antibody optimization and allows for easy separation of unbound reagents [19]. In *indirect* ELISA, an unconjugated antibody binds to the target antigen. Horseradish peroxidase- (HRP-) or alkaline phosphatase- (AP-) conjugated secondary antibodies provide sensitive detection of antigen by providing enzymatic reaction upon the addition of a colorimetric substrate, such as TMB (3,3′,5,5′-tetramethylbenzidine). As reaction time increases, the color intensity continues to increase, resulting in a linear, amplified signal. A stop solution (typically 1 M H_2_SO_4_) is then added to stop the enzymatic reaction (i.e., with TMB). This produces a stable signal for acquiring absorbance at 450 nm and 570 nm wavelengths. The 450 nm reading is directly related to the amount of antigen present, while the 570 nm reading measures the optical density (OD) attributable to plate defects and is used to correct the 450 nm reading. The *competitive* ELISA is often used to show antibody/signal specificity towards antigen with excess unlabeled antigen introduced to “compete” for labeled antibody binding. The *sandwich* ELISA has been applied to give sensitive results as it enriches the antigen through a “capture step” and has served as the basis for the development of “in-solution” quantification, which improves reaction kinetics and avoids issues associated with mass transport. ELISAs are *quantitative* as the amount of antigen is proportional to the amount of standard at any given point along the standard curve. Standard curves often exhibit nonlinear trends and require complex models to represent the best fit line [20]. However, ELISAs are also *semiquantitative* in that a calibrator is used to equate antibody-defined reactivity back to a reference standard [14, 21].

### 4.1. Limitations of ELISA

The various advantages and limitations of ELISAs are provided in Table 1. ELISA advantages include an easy-to-implement and straightforward format, the low cost of reagents and equipment, high sensitivity and specificity (dependent on antibody pair), and a wide variety of applications for detection of proteins, cytokines, and antibodies in serum, plasma, or urine. Certain limitations such as large sample volumes (50-100 *μ*L per well), high variability, and a narrow dynamic range (2-3 orders of magnitude) [2] are inherent to the use of ELISA. ELISAs require a good degree of technical expertise when optimizing experimental conditions and identifying sources of error. Additionally, denatured proteins with intact epitopes are detected by ELISA; therefore, ELISAs are unable to differentiate active and nonactive forms of proteins or biological activity [22]. Unfortunately, most ELISA protocols range from a few hours to days, which is not realistic in a clinical setting. With continued demand for clinical diagnostics for cancer biomarkers, new immunoassay development has transitioned away from conventional ELISA types to paramagnetic or flow cytometry separation paired with chemiluminescent or fluorescent detection, to implement speed and multiplexing techniques, which we present below.

## 5. Chemiluminescent Immunoassays Using Microplates and Microparticles

Chemiluminescent immunoassays (CLIAs) address some of the challenges faced with conventional ELISA. CLIAs have a wide dynamic range (6-7 orders of magnitude), implement shorter protocols (antibody-antigen complex forms more readily due to free mass transfer), do not involve an external light source [23], and require a similar sample volume size as ELISA. An overview of the main advantages and limitations of the established methods discussed is provided in Table 1.

CLIAs use opaque, white microplates and polystyrene or paramagnetic particle “beads” for more sensitive detection. In an indirect or sandwich CLIA, an AP- or HRP-conjugated secondary antibody is detected with an AMPPD (adamantyl 1,2-dioxetane aryl phosphate) or luminol substrate, respectively [24]. Lumi-Phos 530 (Lumigen, Beckman Coulter) is a luminol-based reagent that is approximately 10,000 times more sensitive than colorimetric substrates [25]. The light generated from the chemical reaction between the enzyme and substrate is measured using a photomultiplier in relative light units (RLUs). Like in ELISA, the amount of antigen is proportional to the amount of calibrant/standard at a given point along the standard curve. Plate washers for these assays utilize magnets to separate paramagnetic particles from solution and avoid particle loss. A more in-depth understanding of the “insolution” bead-based assays that are amenable to “homebrewed” antibody use is provided in Section 6 and is the basis of the automated clinical analyzer platform described in Section 7. The application of bead-based antibody detection has been further strengthened by the multiplexing technologies described in Section 8.

## 6. Paramagnetic Particle Considerations

Today, all major diagnostic companies utilize paramagnetic particles (PMP) when designing biomarker assays. These assays fit their automated PMP platforms and provide a universal approach. Important considerations when designing a paramagnetic particle assay include particle surface, size, percent ferrite, material content, and antibody concentration [26]. Paramagnetic particles typically range from 100 nm to 10 *μ*m. When converting an ELISA assay to a PMP assay, a greater number of small particles is required as compared to larger particles to achieve the same surface-area-to-volume ratio used in the ELISA, to keep the antibody concentration on the surface of the particles constant. Most paramagnetic particles are comprised of a polymer and iron matrix and come in different forms. These forms include particles with iron evenly distributed or encapsulated in a polymer matrix [27]. The particle's ferrite content influences the degree of separation between the particles and the solution. A high percent ferrite content results in faster magnetic separation but increases the chances of leaching and interference with the enzyme. Small particles may undergo higher degrees of exposure to the magnetic field due to the influence of drag time and longer separation times due to smaller diameters [28]. Longer separation time can result in agglomeration, which is often irreversible. Lastly, the selected surface chemistry influences how antibody binding to the particle will be achieved (described below). Therefore, conceptualizing the final application and selecting the starting particle and the conjugation chemistry may perhaps be as important as having a validated antibody pair.

### 6.1. Conjugating to Create “Homebrewed” Particles

**Figure 3:**
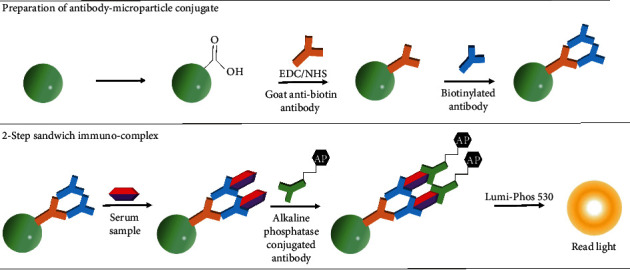
Magnetic antibody-microparticle conjugation. A strategy to prepare a universal magnetic microparticle that can be conjugated to any biotinylated capture antibody has been devised following the methodology set forth in Beckman Coulter literature.

Antibodies can be coupled to a variety of bead surfaces, including modified, preactivated, or bioactivated [29]. A common modified surface chemistry (carboxyl terminated, carboxyl beads) is often used to couple antibody to the bead using EDC/NHS (1-ethyl-3-(3-dimethylaminopropyl)-carbodiimide/N-hydroxysuccinimide), which forms a covalent amide bond. This direct conjugation produces a stable bead; however, carboxyl coupling can be susceptible to agglomeration. Another approach is to create a universal bead (that has a shell of anti-biotin antibody), which can then bind any selected biotinylated capture antibody. This design improves the sensitivity of antigen capture and is applied in Beckman Coulter's Access® Monitor strategy (shown in Figure 3) [30]. Epoxy/tosyl beads are preactivated and can easily be coupled to the antibody using a neutral or alkaline pH but are generally more expensive. Bioactivated bead surfaces include streptavidin-, biotin-, protein G-, and protein A-coated particles. Although streptavidin-coated particles are widely used (as the streptavidin-biotin bond is one of the strongest covalent bonds known in biochemistry), precautions should be taken, as endogenous biotin in the sample may bind, giving potential for false-positive results (a warning given in many streptavidin-based CLIA laboratory assays). No matter what chemistry is chosen, checkerboard assays should be performed to ensure that the bead concentration used and the amount of coupled capture antibody does not limit antigen binding. It is also important to ensure that the enzyme conjugation or detection antibody labeling methods are robust, and the stability of the paramagnetic particle should be investigated over time. Bead recovery after antibody coupling is determined using a hemacytometer or automated cell counter [31] prior to determining the antibody coupling efficiency, which is quantified by creating a dose-response curve for the particles using a fluorescent-labeled secondary antibody (i.e., phycoerythrin (PE)) [31].

### 6.2. Beckman Coulter Conventional and “Orientation-Specific” Antibody Coating

Beckman Coulter has created a universal PMP that can be conjugated with a multitude of biotinylated primary antibodies in individual (singleplex) biomarker immunoassays (currently 54 offerings). In this approach, a goat anti-biotin antibody is coupled to a carboxyl paramagnetic particle using EDC/NHS chemistry (conventional binding). Any unbound carboxyl groups are blocked to prevent nonspecific binding. The goat anti-biotin conjugated particles are then incubated with a biotinylated capture antibody and the sample or standard (antigen) in a single step (Figure 3). This approach allowed Beckman Coulter to produce a universal bead that permits binding to any of their primary biotinylated antibodies. The addition of an alkaline phosphatase-conjugated (detection) antibody completes the 2-step sandwich immunocomplex. Detection of RLUs is measured using a luminometer following the addition of the Lumi-Phos 530 substrate.

**Figure 4:**
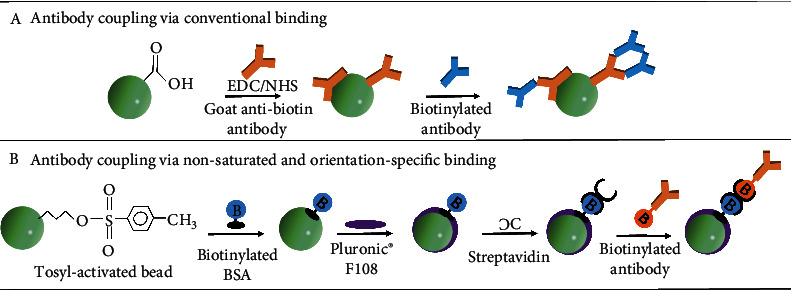
Antibody conjugation to the surface of the paramagnetic particle: (a) antibody conjugation using conventional antibody methods; (b) “orientation-specific” antibody conjugation using Beckman Coulter's 3-layer antibody coupling approach.

Beckman Coulter has proposed an alternate coupling approach to address concerns associated with conventional binding. When an antibody is bound conventionally, it can bind the surface in any direction (Figure 4(a)), which in turn can influence antigen binding [32]. However, using a nonsaturated approach, one can introduce “orientation-specific” binding (Figure 4(b)). Beckman Coulter has patented a 3-layer binding approach for nonsaturated and “orientation-specific” binding; however, we did not see its application in their current biomarker diagnostic assays. This approach (US8518714B2-binding surface for affinity assays) involves the covalent binding of biotinylated BSA to tosyl-activated paramagnetic particles. Unbound sites are then blocked with Pluronic® F108. Streptavidin is coupled to the biotinylated BSA via lysine-based conjugation (amine reaction) [33] followed by addition of the biotinylated capture antibody [34]. Studies demonstrate significant improvement in assay signal-to-noise and dynamic range for nonsaturated particles compared to their conventionally coated counterpart (goat anti-biotin microparticles) (Figure 4(a)). The lower background is the result of enhanced surface blocking and improved binding efficiency.

## 7. Automated Biomarker Assays

The major automated platforms and assays for serum biomarkers were developed by Beckman Coulter, Abbott, Fujirebio Diagnostics, and Roche Diagnostics. These robust serum-based assays provide sensitive detection with high reproducibility and a low coefficient of variation (CV < 5%) [35, 36]. The robustness of an assay is validated by assessing linearity, the limit of blank/detection/quantification, and spike recovery [37, 38]. Automated platforms are equipped with injectors to accurately deliver set volumes. Shortened protocols provide results in as little as 25 minutes. Each vendor has its own unique assay and detection method, whether it is flow cytometry, luminometer, or charge-coupled device (CCD) camera; therefore, patient sample testing must be performed on the same platform to produce comparable results, as each platform may have different cutoffs for the same biomarker [39]. Beckman Coulter has developed over 50 biomarker assays for different diseases, each utilizing its own standard (calibrant) and universal paramagnetic particle design. Their platform (UniCel DxI 800 Access Immunoassay System) and other sequential injection analysis (SIA) machines eliminate the risk of human error and sources of technical variability by automating almost every step [40].

Automated PMP assays are ideal in the hospital clinical chemistry lab environment due to the large volume of serum sample tests that are ordered by hospital physicians. In the clinic, multiple blood vacutainers are drawn to provide the required serum volume (typically 500 *μ*L-2 mL) for each automated immunoassay (enough to perform the assay twice). Thus, patient serum is not a limiting factor. However, in the research lab setting, precious/small serum samples (typically 100-500 *μ*L per sample) usually limit the number of replicates and biomarker assays that can be performed. Therefore, incorporating a multiplexing approach into the PMP format is very attractive and perhaps essential for the identification/study of new biomarkers.

## 8. Multiplexing

### 8.1. Luminex Assay

**Figure 5:**
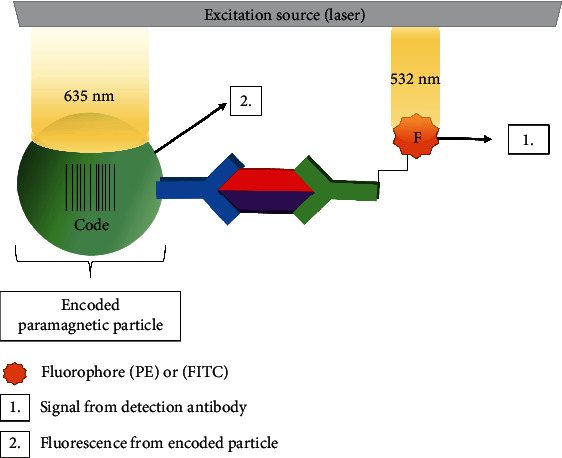
Biomarker detection using flow cytometry. The Luminex strategy detects multiplexed biomarkers using dual wavelengths. The two fluorescent dyes are excited at a wavelength of 635 nm, and the fluorophore is excited at 532 nm. The fluorescent light emitted from the bead is detected at two separate wavelengths, corresponding to a specific biomarker, while the signal produced from each fluorophore is detected at another wavelength.

Luminex has developed a biomarker multiplexing platform that uses fluorescent detection. Currently, Luminex beads support the detection of 96 validated antibody pairs. Cross-reactivity between antibody pairs has been assessed by either Luminex or Luminex partners [41]. The main benefits of this technology are that multiple biomarkers can be tested simultaneously in a small sample volume (12-25 *μ*L), statistical comparisons are efficiently made by counting (10-1000) positive beads for each biomarker, and time is saved. Luminex's paramagnetic particles are constructed with different ratios of red and yellow fluorescent dyes that serve to identify each particle as a “barcode”. A unique capture antibody is conjugated to each separate barcoded particle for capture, and phycoerythrin (PE) or fluorescein isothiocyanate- (FITC-) conjugated antibody is added for quantification of target molecules (Figure 5). A mixture of all the desired capture beads is used to “multiplex” detection of each sample. FITC detection is not as sensitive as chemiluminescent or colorimetric (HRP/TMB) assays [42]. However, it is stable and provides a direct label that does not require an enzymatic reaction. PE and FITC are measured at their respective wavelengths with readings/signals tallied for multiple beads and biomarkers identified by their unique barcodes (depicted in Figure 5) [31]. Validated hardware and statistical approaches have made Luminex technology a standard for pathogen detection. For example, the Luminex xTAG® Gastrointestinal Pathogen Panel is an analytically and clinically validated assay for the Luminex detection system(s). In addition, Millipore's various research use only Milliplex panels are available and include the Human Circulating Cancer Biomarker Magnetic Bead Panel, Human Cancer Biomarker Panel, and Human Chemokine Panel [43].

### 8.2. Quanterix™ Single-Molecule Array (SiMoA)

Quanterix's unique multiplexing technology, known as digital ELISA or single-molecule array (SiMoA), combines the Singulex Erenna and Luminex platform concepts. This specially designed assay is 1000 times more sensitive than ELISA and has a dynamic range of 10^4^ and is capable of multiplexing up to 10 analytes [44]. The SiMoA assay is similar to Luminex's assay in that a specific capture antibody is coupled to dye-coded paramagnetic particles. In multiplex assays, paramagnetic particles coded with different specific capture antibodies are pooled to simultaneously detect multiple analytes in 100 *μ*L [45] of serum samples. Serum samples are incubated with an excess number of antibody-coated particles compared to the number of target molecules; therefore, either one protein molecule or zero binds to the bead [46]. A mixture of biotinylated antibody is added, followed by streptavidin *β*-galactosidase, which completes the immunocomplex. Next, the fluorescent substrate resorufin *β*-D-galactopyranoside is added, and the particles are separated into individual reaction wells, which are fabricated based on the bead's diameter (2.7 *μ*m) [47, 48]. Single immunocomplexes are detected using a CCD camera to distinguish wells containing a single labeled molecule (fluorescent signal) from those not containing a single labeled molecule (no fluorescent signal) [49]. The total concentration of a protein present in the sample is expressed as a percentage of beads carrying a protein molecule to the total number of beads. This sensitive technology allows for the detection of single molecules with a limit of detection (LOD) of subfemtomolar levels, which is 1000 times greater than Luminex's LOD (picomolar).

### 8.3. Millipore Single-Molecule Counting

Millipore's single-molecule counting (SMC™) addresses a concern that high signals may be associated with the presence of a nonspecifically bound detection antibody. In the assay, biotinylated antibody is coupled to streptavidin particles and forms an antibody-antigen complex when incubated with sample and fluorescently labeled detection antibody. The microparticles are then transferred to a new 96-well plate after washing away the unbound detection antibody. The addition of elution buffer (4 M urea) releases the detection antibody from the microparticles by disrupting the antigen-antibody interaction [50]. The detection antibody is separated from the elution buffer and detected as a free-floating fluorochrome. As the fluorochrome transits a capillary, a laser causes its fluorescent emission in an interrogation space for “avalanche” photodiode detection. This special type of photodiode operates at a higher reverse bias, which results in an internal gain to increase the effective responsiveness and sensitivity of the device [51]. Multiplexing is implemented by using antibodies specific to each biomarker, labeled with different fluorescent dyes; therefore, a limited number of antibody pairs may be multiplexed in order to minimize cross-reactivity. Ideally, selected dyes have a narrow bandwidth and possess different ranges of emittance. Multiple detectors can then be used to differentiate multiple dyes using a variety of filters and diffraction gratings. One disadvantage of the technology is that quenching may occur for doubly labeled molecules, resulting in loss of fluorescent signal.

### 8.4. BD Biosciences Cytometric Bead Array (CBA)

BD Biosciences offers the BD™ CBA assay, a multiplexing assay for the detection of up to 30 proteins in a 25-50 *μ*L sample volume, and requires less sample dilution as compared to ELISA. Lyophilized standards and validated antibody pairs primarily for chemokine and inflammatory mediators are offered. Beads are encoded with varying intensities of one fluorescent dye and are coupled to specific capture antibodies using sulfo-SMCC chemistry [52]. Sample, beads, and fluorescent labeled- (PE) detection antibodies are incubated to form sandwich immunocomplexes, which are detected using a BD FACS™ flow cytometry system or any flow cytometer capable of outputting data in the flow cytometry standard (FCS) data file format. The company offers a wide variability of kits for multiplexing cytokines such as CBA Flex Sets and CBA enhanced sensitivity kit with detection limits of 10 pg/mL and 0.274 pg/mL, respectively. The different bead sets within each kit are differentiated by evaluating a fluorescence parameter and two size discriminators using fluorescent and scatter signals [53]. These kits have been used to multiplex the following six cytokines: IL-2, IL-4, IL-5, IL-10, IFN-gamma, and TNF-alpha [54, 55].

## 9. Clinical Application

The development of monoclonal antibodies has influenced the progression and development of immunoassays. Variability between commercial assays is heavily dependent on the specificity and sensitivity of the capture and detection antibodies, the composition of diluent, and pH [37]. These are important considerations in multiplex assays, which are exemplified in the literature by the reported binding affinities of different antibodies at a specific pH and salt concentration [56, 57]. Sample diluents are used to minimize interference or “matrix effect” observed in serum samples. In PMP assays, magnetic separation aids in the removal of interfering components from the targeted analytes and reduces the nonspecific binding of detection antibodies with capture antibodies [58, 59]. Another technique to reduce cross-reactivity and nonspecific binding includes sequential protein capture. This method involves coupling beads with different capture antibodies, which are then sequentially incubated with a sample. Each type of antibody-coupled bead is incubated with the sample and is separated from the sample using a magnet before incubating the sample with the next antibody-coupled bead (different capture antibodies) [46]. In this case, each type of antibody-coupled bead is processed individually, so it can be incubated with its corresponding detection antibody.

Bead-based assays are commonly used for the detection of hormones, cytokines, growth factors, tumor biomarkers, and antibodies. Luminex's multiplexing assay has been used to identify proteins associated with the efficacious treatment of cancer with drugs, such as colorectal cancer with cetuximab, and to differentiate early- and late-stage ovarian cancer [60]. Other bead-based assays, such as the SiMoA, have been applied to detect prostate-specific antigen (PSA) for the detection of prostate cancer [61, 62] and inflammatory cytokines at low fg/mL or sub-fg/mL levels. Paramagnetic particle assays exhibit faster phase kinetics, larger surface area, and lower limits of quantification yet are often compared to ELISA before obtaining clinical acceptance [53].

## 10. Conclusions

Growing demand for validated paramagnetic particle assays is improving the detection of biomarkers in serum. Although ELISA is a widely used technique, it does not provide the dynamic range and robustness of the newer PMP enzymatic and fluorescent assays. ELISA does continue to have its place as a cost-effective R&D tool, which can be translated to paramagnetic particle assays. CLIAs provide a wide dynamic range of six to seven orders of magnitude and require a similar sample volume as compared to ELISA. Particle size, percent ferrite, surface chemistry, matrix, wash optimization, antibody pair selection, antibody concentration, and calibrator use are all factors that influence the development and use of paramagnetic particle assays. Paramagnetic particles are utilized by all major developers of automated assays/platforms for the detection of serum biomarkers in approved clinical diagnostic immunoassays. While Beckman Coulter has developed multiple singleplex biomarker assays using their universal goat anti-biotin bead approach, Luminex and Millipore have focused on multiplexing biomarkers. Although multiplexing may be desired in the research setting where serum samples are often limiting, it is often inaccessible due to the required platform instrumentation costs. Therefore, the indirect and sandwich ELISA techniques continue to have their place, and sequential capturing may serve the purpose of limited multiplexing goals. Improvements to ELISA include chemiluminescent detection and paramagnetic particle construction. These principles can be incorporated into the research lab setting to improve assay sensitivity, avoid the need for sample dilution through a wider dynamic range, and reduce incubation time.

## Figures and Tables

**Table 1:** Advantages and limitations of current immunoassay methods.

Method	Advantages	Limitations
ELISA (colorimetric)	(i) Simple and easy to implement (ii) Cost-effective (iii) High sensitivity and specificity (iv) Wide variety of applications (v) Protocol conditions can be applied to bead-based assay development	(i) Immobilization on solid surface (ii) Sample volumes of (50-100 *μ*L per well) (iii) Protocols range from a few hours to days (iv) Narrow dynamic range (2-3 orders of magnitude) (v) High variability (vi) High nonspecific absorption (vii) Unable to multiplex (singleplex assay format)

CLIA (bead-based, paramagnetic, luminescence)	(i) Short protocols (ii) High sensitivity and robustness (iii) Wide dynamic range (6-7 orders of magnitude) (iv) High stability of reagents (v) In-solution detection	(i) Sample volumes of (50-100 *μ*L per well) (ii) High cost of equipment (luminometer) (iii) Complex reaction kinetics (nonlinear response) (iv) Emission intensity dependent on time (v) Influenced by environmental factors (e.g., temp., light, and pH) (iv) Unable to multiplex (singleplex assay format)

Bead Array Kits (BD™ CBA assay)	(i) Large surface area-to-volume ratio (ii) Small sample volumes (12-25 *μ*L) (iii) Medium to high throughput (multiplex) (iv) High signal-to-noise ratio (v) High sensitivity/low variability	(i) Stability and emittance of fluorophore dependent on pH (ii) Potential for antibody cross-reactivity in multiplex assays (iii) High cost of instrumentation (dedicated system or flow cytometer) (iv) Antibody clones unknown in commercial kits (v) Autofluorescence from interfering components in serum

Bead-based multiplexing technologies (Luminex, Quanterix/SiMoA, Millipore SMC™)	(i) Minimize nonspecific binding (ii) Medium to high throughput (multiplex) (iii) High sensitivity (low limit of detection) (iv) Wide dynamic range (4-6 orders of magnitude)	(i) Unique equipment for each type of analysis platform (ii) Tradeoffs between number of possible multiplexed (iii) Limited applications (proteins, nucleic acids, and small molecules) (iv) Fluorophores may interfere with antibody-antigen binding

## Abbreviations

MRI: Magnetic resonance imaging
RIA: Radioimmunoassay
EIA: Enzymatic immunoassay
ELISA: Enzyme-linked immunosorbent assay
MDC: Minimum detectable concentration
ROC: Receiver operating characteristic
SN: Sensitivity
SP: Specificity
RUO: Research use only
WHO: World Health Organization
NIBSC: National Institute for Standards and Controls
GI: Gastrointestinal
AUC: Area under the curve
MRF: Mucin-rich fraction
HRP: Horseradish peroxidase
AP: Alkaline phosphatase
AMPPD: Adamantyl 1,2-dioxetane aryl phosphate
TMB: 3,3′,5,5′-Tetramethylbenzidine
OD: Optical density
CLIA: Chemiluminescent immunoassay
RLU: Relative light unit
PMP: Paramagnetic particle
PE: Phycoerythrin
EDC: 1-Ethyl-3-(3-dimethylaminopropyl)-carbodiimide
NHS: N-Hydroxysuccinimide
BSA: Bovine serum albumin
CV: Coefficient of variation
CCD: Charge-coupled device
SIA: Sequential injection analysis
FITC: Fluorescein isothiocyanate
SiMoA: Single-molecule array
LOD: Limit of detection
SMC: Single-molecule counting
CBA: Cytometric bead array
FCS: Flow cytometry standard
PSA: Prostate-specific antigen
R&D: Research and development.
